# The 3-*O* sulfation of heparan sulfate proteoglycans contributes to the cellular internalization of tau aggregates

**DOI:** 10.1186/s12860-022-00462-1

**Published:** 2022-12-24

**Authors:** Andreia Ferreira, Ines Royaux, Jian Liu, Zhangjie Wang, Guowei Su, Diederik Moechars, Nico Callewaert, Louis De Muynck

**Affiliations:** 1 Janssen Research & Development, a Division of Janssen Pharmaceutica N.V, 2340 Beerse, Belgium; 2 VIB Center for Medical Biotechnology, Ghent, Belgium; Department of Biochemistry and Microbiology, Ghent University, Ghent, Belgium; 3 Division of Chemical Biology and Medicinal Chemistry, Eshelman School of Pharmacy, University of North Carolina, Chapel Hill, NC 27599 USA; 4 Glycan Therapeutics, LLC, 617 Hutton Street, Raleigh, NC USA

**Keywords:** Tau uptake, Heparan sulfates proteoglycans, 3-O sulfation, HS 3-O sulfotransferase 1, Tau-heparan sulfates interaction

## Abstract

**Background:**

Considering the high correlation between the functional decline in Alzheimer’s disease (AD) and the propagation of aggregated tau protein, many research efforts are focused on determining the underlying molecular mechanisms of tau spreading. Heparan sulfate proteoglycans (HSPGs) were reported to mediate cellular uptake of tau aggregates. Specifically, the heparan sulfates (HS) sulfation plays a critical role in the interaction of HSPGs with aggregated tau. HS can be *N*−/2-*O*/6-*O*- or 3-*O*-sulfated, some of which have been reported to take part in the interaction with tau aggregates. However, the role of the 3-*O* sulfation remains enigmatic.

**Results:**

Here, we studied the contribution of HS 3-*O* sulfation in the binding and cellular uptake of tau aggregates. We observed reduced tau aggregates uptake in absence of 3-*O* sulfation or when outcompeting available cellular 3-*O* sulfated HS (3S-HS) with antithrombin III. The lack of HS3ST1-generated HS products in the *HS3ST1*^−/−^ cells was further corroborated with an LC-MS/MS using ^13^C-labeled HS calibrants. Here, we showed that these functional changes can be explained by a higher affinity of aggregated tau to 3S-HS. When targeting tau aggregates with 3-*O* sulfation-containing HS, we observed an increase in inhibition of tau aggregates uptake.

**Conclusions:**

These data indicate that HS 3-*O* sulfation plays a role in the binding of tau aggregates and, thus, contributes to their cellular uptake, highlighting a potential target value to modulate tau pathogenesis.

**Supplementary Information:**

The online version contains supplementary material available at 10.1186/s12860-022-00462-1.

## Background

Alzheimer’s disease (AD) is the most common form of dementia with 115 million people to be expected to have AD by 2050 [1]. One of the hallmarks of AD is the presence of neuronal inclusions called neurofibrillary tangles (NFTs) composed of hyperphosphorylated and aggregated tau protein, presented under the form of paired helical filaments (PHFs) [2, 3]. In the brain of AD patients, tau pathology follows a spatiotemporal pattern of distribution, starting in a confined region of the brain and gradually spreading throughout the brain with the course of the disease, a phenomenon at the basis of the Braak staging [4]. Strikingly, the density and neocortical load of NFTs show a high correlation with the progressive neuronal degeneration [5, 6] and the severity of AD [4, 7]. Tau propagation is believed to occur by the release of a pathological tau seed from a donor neuron and subsequent uptake by a recipient neuron, where it will induce tau pathology by recruiting non-pathological tau monomers, a phenomenon defined as prion-like propagation of tau pathology [8, 9]. Such seeding mechanism is hypothesized to underlie the progression of pathology observed for other proteins associated with neurodegenerative diseases, such as amyloid-β [10, 11] and α-synuclein [12, 13]. Importantly, this pathology propagation occurs within anatomically connected regions, suggesting a transsynaptic spread of the pathology rather than mere diffusion [14], although such hypothesis has not been fully demonstrated yet. This suggests that, if propagation of pathology between neurons underlies disease progression, interruption of such process holds promise for the development of disease-modifying therapies, for example, by modulating the uptake of tau seeds.

Earlier work has put forward a role for heparan sulfate proteoglycans (HSPGs) as mediators of seeds uptake and propagation [15]. HSPGs are made of carbohydrate polymers of repeating disaccharides building blocks, − consisting of (N-acetylated−/sulfated) glucosamine (GlcNAc) and a hexuronic acid (glucuronic or iduronic acid), − linked to a core protein. They are responsible for cell signaling and tissue distribution of molecules such as growth factors and morphogens [16]. The heparan sulfate (HS) chains undergo a series of modifications in the Golgi apparatus, one being the sulfation. There are four families of heparan sulfate sulfotransferases, classified according to the site they modify: 2-*O*-sulfotransferases, that modify the 2-OH position of the hexuronic acid group, and N-deacetylase/N-sulfotransferase, 6-*O*-sulfotransferase and 3-*O*-sulfotransferase that modify the N-acetyl, 6-OH and 3-OH position, respectively, of the glucosamine group. The different sulfotransferase families consist of different isoforms that are involved in the formation of HS motifs with a distinct temporal and spatial pattern. This accounts for highly heterogeneous and unique sulfation patterns that mediate the specific interactions of HSPGs with their ligands [17].

Strikingly, early studies have reported immunoreactivity of NFTs with HSPGs in AD brain tissues [18, 19]. In vitro studies demonstrated that sulfated glycosaminoglycans can promote the fibrillation of tau into filaments [20], and a correlation between HS sulfation and abnormal tau phosphorylation [21] and aggregation [22] has been observed. A lot of focus has been given into investigating the different sulfotransferases and HS sulfation types that can be involved in tau-HS binding and cellular internalization. Using a myriad of (bio) chemical and genetic approaches, it has been reported that 2-*O* sulfation is not essential for HS-tau binding nor cellular uptake of both monomeric and aggregated tau [23, 24]. While 6-*O* sulfation has been reported to play a role in the uptake and binding of HS to both monomeric and aggregated tau and consequent seeding of aggregated tau [24, 25], *N*-sulfation seems to contribute to the internalization, binding, and seeding of HS to aggregated tau [24], but not to the monomeric tau binding to HS [25]. Recently, 3-*O* sulfation was also reported to engage in the interaction of HS with monomeric tau [26].

Three isoforms of the heparan sulfate 3-*O* sulfotransferases (*HS3ST2*, *HS3ST4* and *HS3ST5*) are predominately expressed in AD-relevant brain regions [27–29], with *HS3ST2* and *HS3ST4* mRNA levels being reported to be significantly increased in the AD hippocampus [21]. Furthermore, heparin, an HS with high degree of 3-*O* sulfation [30], has been shown to not only promote tau aggregation but also its phosphorylation [22]. Lastly, the *HS3ST2* isoform was reported to be critical for abnormal tau phosphorylation [21]. Altogether, these data point towards a role of *HS3STs* and 3-*O* sulfated HS (3S-HS) in tau pathology. However, it remains unclear what role the HS 3-*O* sulfation plays in the interaction with aggregated tau.

In this work we assessed the contribution of 3-*O-*sulfated HS in the cellular uptake of tau preformed fibrils (PFFs). For that, we used the HCT-116 cell line as we only detected the expression of one *HS3STs* isoform, the *HS3ST1*. We demonstrated that modulation of 3-*O* sulfation in HCT-116 cells by either targeting 3S-HS with antithrombin III (ATIII) protein, or genetically by KO of the only *HS3ST* isoform expressed by this cell line, *HS3ST1,* reduced the uptake of tau PFFs, a phenotype that could be rescued upon *HS3ST1* overexpression. Using synthetic oligosaccharides with defined sulfation patterns, we also observed that tau PFFs bind with a higher affinity to 3S-HS and that targeting tau PFFs with the same synthetic 3S-HS oligosaccharides resulted in a higher inhibition of the uptake in HCT-116 cells. Altogether, these results suggest, for the first time, a role of *HS3STs* and, thus, 3-*O* sulfation in the binding and uptake of aggregated tau protein.

## Results

### The uptake of tau PFFs is HSPGs-mediated in HCT-116 cells

**Fig. 1:**
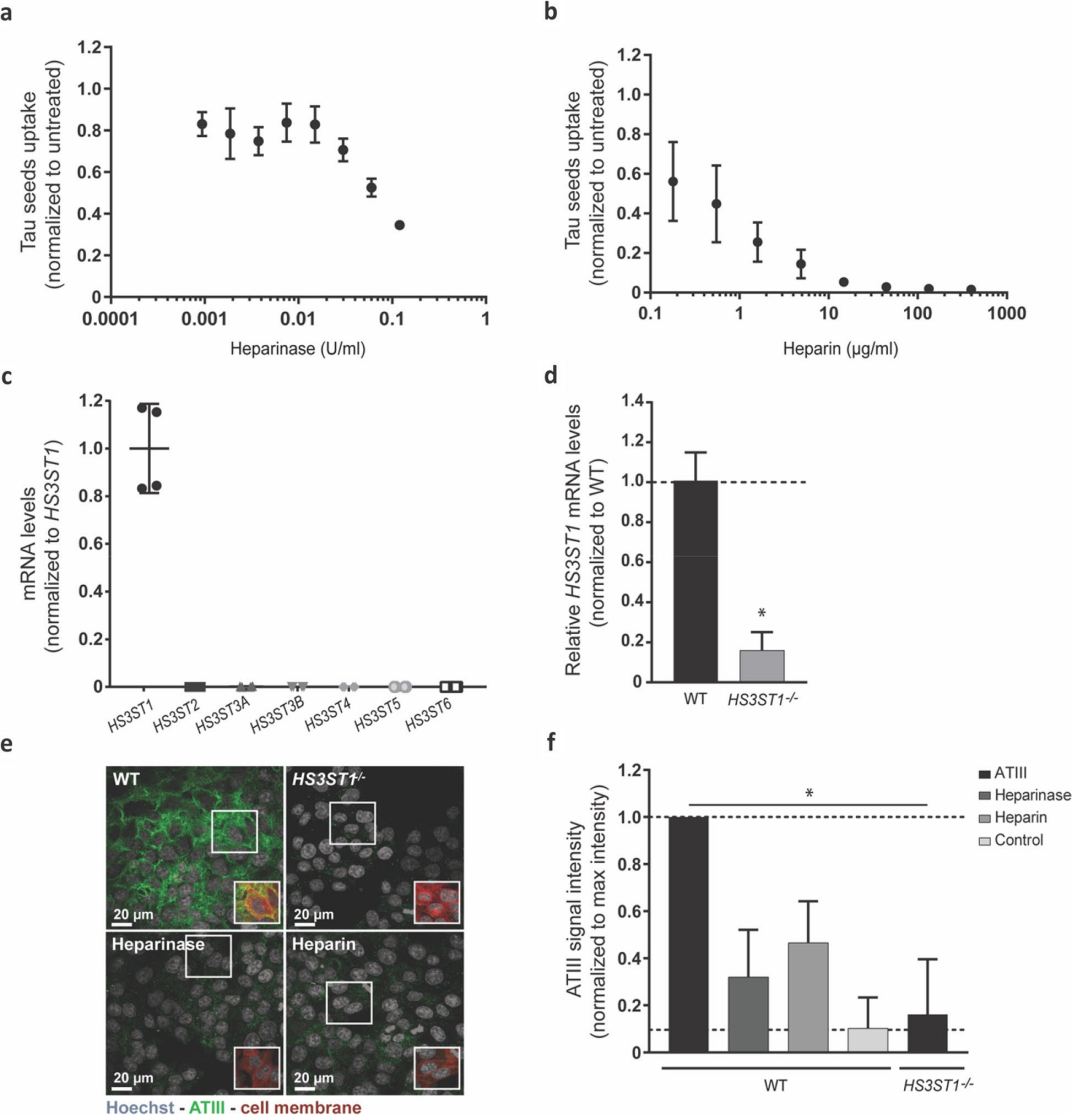
Validation of the *HS3ST1*^−/−^ HCT-116 cell line with HSPGs-mediated uptake of tau PFFs. **a**, **b** Treatment of HCT-116 cells with either heparinase (**a**) or heparin (**b**) results in a reduction of tau-AF488 seeds uptake, confirming a role for a HSPGs-mediated uptake mechanism (**c**) The mRNA expression profile of *HS3STs* in HCT-116 cell line, analyzed by qPCR (TaqMan), shows that only the *HS3ST1* isoform is expressed by this cell line. **d** mRNA expression levels of the *HS3ST1* isoform in WT and *HS3ST1*^*−/−*^ cells by qPCR (TaqMan), demonstrate an 80% reduction of *HS3ST1* mRNA levels in the *HS3ST1*^*−/−*^ cell line when compared to WT (Mann-Whitney test between WT and *HS3ST1*^*−/−*^ cells, * *p* < 0.05). **e** Immunostaining of exogenous ATIII bound to cell surface HS in *HS3ST1*^*−/−*^ cells (top right) and WT (top left, bottom left and right) showing reduced ATIII signal in *HS3ST1*^*−/−*^ cells, as well as WT cells pre-treated with either heparinase or heparin (bottom panel). Frames show the cell membrane staining, demonstrating the overlap between the ATIII signal and the cell membrane. For image acquisition, 13 fields with 4 z-stacks were analysed with around 2000 cells per field. **f** quantification of ATIII signal intensity normalized to background levels revealing a significant reduction of ATIII signal in *HS3ST1*^*−/−*^ cells, comparable to the control condition (no exogenous ATIII added) (Kruskal-Wallis with Dunn’s post hoc test to compare WT cells ATIII intensity to the other conditions, * *p* < 0.05). Data represented as mean ± SD of 3 or 4 independent experiments (biological replicates)

To confirm previous reports of an HSPGs-mediated cellular internalization of tau PFFs [15], HCT-116 cells were treated with either heparinase III (16 h), − specific for the cleavage of cell surface HS, − or heparin (3 h), − a competitor for the HS binding sites on the tau PFFs, − before a 24 h incubation with fluorescently labeled tau PFFs (tau-AF488). Uptake of tau-AF488 was visualized via live-cell confocal microscopy. Treatment with heparinase (Fig. 1a) and heparin (Fig. 1b) resulted in a dose-dependent reduction in the cellular uptake of tau-AF488 PFFs, with 65% reduction with heparinase III (0.12 U/ml) and 100% inhibition of uptake upon treatment with heparin (130 μg/ml). Together, these results demonstrate that both enzymatic digestion of cell surface HS and blocking of HS binding sites on tau PFFs impact uptake, confirming that tau PFFs internalization is meditated by HSPGs.

### Modulation of 3-*O* sulfation levels results in reduced uptake of tau PFFs

To assess the role of 3-*O* sulfation on the uptake of tau PFFs, *HS3STs* expression was abolished in HCT-116 cells using CRISPR/Cas9 technology. The uptake of tau PFFs by the HCT-116 cell line is mediated by HSPGs, as demonstrated before, making it ideal to study the HS structural determinants mediating this mechanism. First, to study 3-*O* sulfation, the *HS3STs* expression profile was determined in the HCT-116 cell line, to guide further KO experiments. Figure 1c shows the mRNA levels of the different *HS3ST* isoforms in the HCT-116 cell line, determined by qPCR, demonstrating that only the *HS3ST1* isoform is expressed in this cell line. Subsequently, CRISPR/Cas9 technology was used to generate a *HS3ST1*^−/−^ HCT-116 monoclonal cell line that was further validated by qPCR. Here, *HS3ST1* mRNA levels in the *HS3ST1*^−/−^ are reduced by 80% when compared to WT HCT-116 (Fig. 1d) also not expressing any of the other *HS3STs* isoforms (data not shown).

Next, a method was developed to assess the levels of 3-*O* sulfation in the *HS3ST1*^−/−^ cells. As antithrombin III (ATIII) is known to interact specifically with 3-*O* sulfated HS [31] (Supplementary Fig. 1), exogenous ATIII protein was added to *HS3ST1*^−/−^ and WT fixed cells, which was then stained and visualized via confocal microscopy. The ATIII signal is reduced in WT cells pre-treated with heparinase III or heparin, confirming that a decrease in cell surface heparan sulfate or competition of ATIII binding to HS with heparin results in the reduction of ATIII intensity (Fig. 1e and f). Interestingly, ATIII signal is highly reduced in the *HS3ST1*^−/−^ cell line (levels similar to background, Fig. 1e and f), substantiating a loss of HS 3-*O* sulfation in the *HS3ST1*^−/−^ cell line, and suggesting that the 20% remaining *HS3ST1* mRNA is not processed into functional protein.

To confirm the reduction in heparan sulfates that contain the ATIII-binding site sequence in the *HS3ST1*^−/−^, an LC-MS/MS analytical method relying on synthetic ^13^C-labeled HS standards was performed [32]. As a control, samples from CHO-K1 cell lines that are reported to weakly express HS3STs and to not produce HS with anticoagulant activity (i.e., do not bind ATIII) [33, 34] – this is demonstrated in Table 1 by the lack of tetrasaccharide 9 detected in the CHO-K1 sample. The raw amount (ng/ml) of the different di/tetrasaccharides and the calibrant recovery yield for the different samples are shared in Tables S1 and S2, respectively. Table 1 demonstrates that the HS content is comparable between the HCT-116 and the *HS3ST1*^−/−^ cells, except for tetrasaccharide **9** (△UA-GlcNAc6S-GlcA-GlcNS3S6S) that demonstrates a 25x increase in the wild-type HCT-116 cell line compared to the *HS3ST1*^−/−^ cell line. These results confirmed the reduction of the *HS3ST1*-modified heparan sulfates in the *HS3ST1*^−/−^ cell line.

**Table 1:** The results of the mass percentage (%) of each disaccharide and tetrasaccharide in the samples

Di/tetra-saccharides		mass percentage (%)					
	CHO-K1		***HS3ST1***^**−/−**^		HCT-116		Fold-change ***HS3ST1***^**−/−**^ / HCT-116
	Mean	SD	Mean	SD	Mean	SD	
**1** △UA-GlcNAc	65,95	0,6	30,7	4,5	39,05	1,2	0,27
**2** △UA2S-GlcNAc	0,95	0,1	0,4	0,0	0,45	0,1	0,13
**3** △UA-GlcNAc6S	2,2	0,3	9,35	0,4	8,65	0,1	−0,07
**4** △UA2S-GlcNAc6S	0	0,0	0,1	0,0	0,1	0,0	0
**5** △UA-GlcNS	20,15	0,1	41,7	5,4	33,8	1,4	−0,19
**6** △UA2S-GlcNS	6,45	0,1	2,55	0,1	2,85	0,1	0,12
**7** △UA-GlcNS6S	1,3	0,1	7,35	0,5	6,75	0,2	−0,08
**8** △UA2S-GlcNS6S	1,75	0,2	2,85	0,2	3,65	0,5	0,28
**9** △UA-GlcNAc6S-GlcA-GlcNS3S6S	0	0,0	0,1	0,0	2,6	0,1	**25**
**10** △UA-GlcNS6S-GlcA-GlcNS3S6S	0,3	0,0	1,45	0,8	1,1	0,4	−0,24
**11** △UA-GlcNS6S-IdoA2S-GlcNS3S6S	0	0,0	0	0,0	0	0,0	0
**12** △UA-GlcNS-IdoA2S-GlcNS3S	0,45	0,1	1,8	0,8	1,4	0,3	−0,22
**13** △UA2S-GlcNS-IdoA2S-GlcNS3S	0,6	0,0	1,65	0,1	1,5	0,3	−0,09

For this experiment, two biological replicates were analysed

**Fig. 2:**
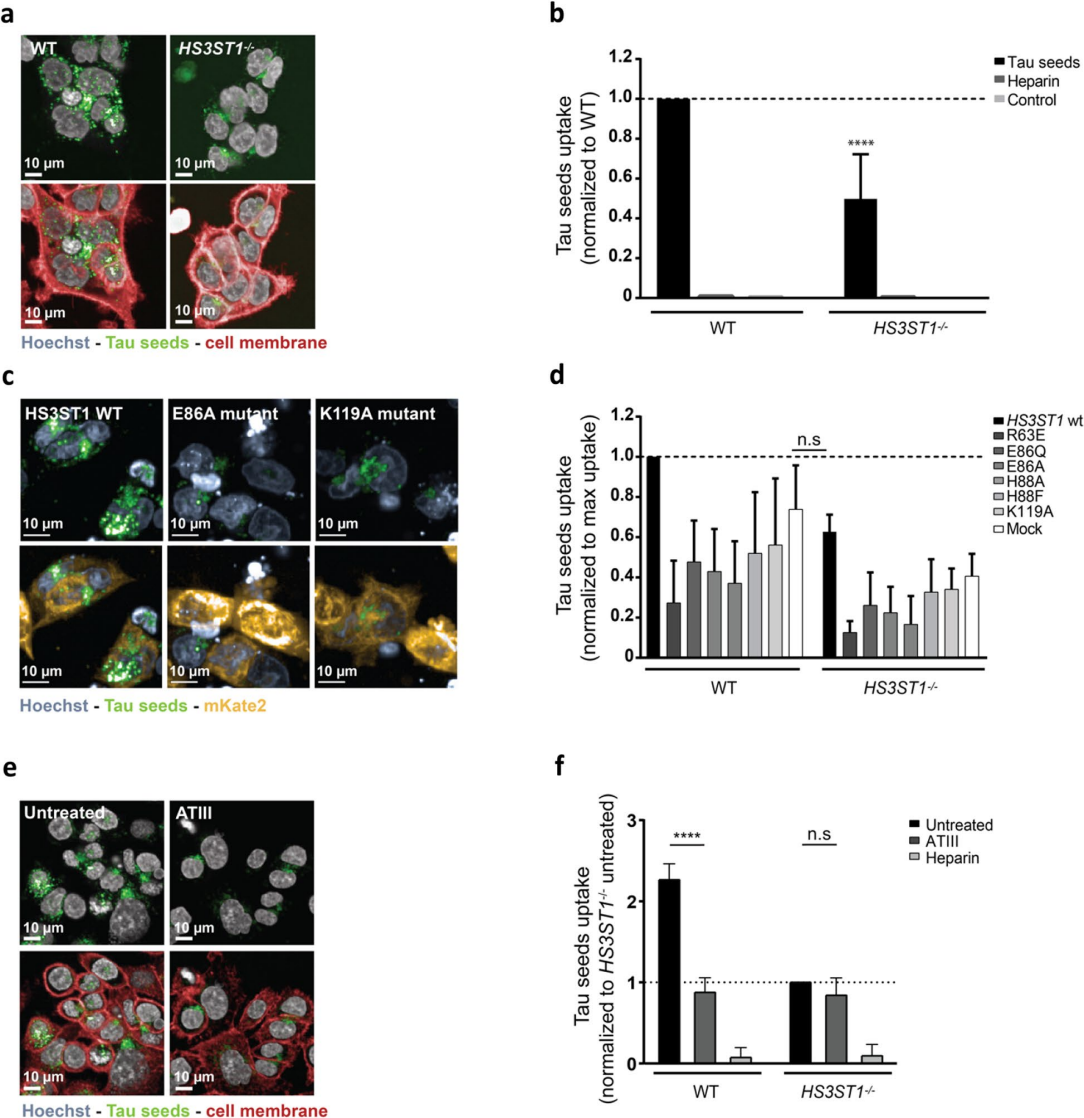
Modulation of 3-*O* sulfation results in decreased uptake of tau-AF488 in HCT-116 cells. **a** Using live-cell microscopy to measure tau-AF488 uptake, it is possible to observe a reduction in the number and intensity of tau-AF488 puncta. **b** Quantification of tau-AF488 uptake normalized to the uptake of WT cells, shows a significant reduction of 50% in tau seeds uptake by the *HS3ST1*^*−/−*^ cells (two-way ANOVA with Sidak’s post hoc against WT cells treated with tau seeds, **** *p* < 0.0001) (**c**) Live-cell microscopy of *HS3ST1*^*−/−*^ cells transfected with either *HS3ST1*-mKate2 WT construct or inactive mutant forms of *HS3ST1*, shows an increase in the number and intensity of tau-AF488 seeds puncta in *HS3ST1* overexpression conditions. The constructs were designed to express mKate2, a fluorescent protein, at the C-terminal to allow the script to quantify the uptake of tau PFFs in transfect cells. The bottom panel shows the images without the mKate2 signal for easier visualization of the tau PFFs puncta (**d**) Quantification of tau-AF488 seeds uptake, normalized to the maximum uptake in WT cells reveals no significant difference in Tau uptake levels between *HS3ST1*^*−/−*^ cells overexpressing *HS3ST1* and WT cells mock transfected, indicating a rescue of uptake upon *HS3ST1* overexpression (two-way ANOVA with Sidak’s post hoc against WT cells mock transfected, n.s *p* > 0.9999). **e** Using live-cell microscopy to measure tau-AF488 uptake in HCT-116 WT cells, it is possible to observe a reduction in the number and intensity of tau-AF488 puncta upon pre-treatment of cells with ATIII protein (**f**) Quantification of tau-AF488 seeds uptake by HCT-116 WT incubated with ATIII show a 60% reduction of tau uptake, which is not observed on *HS3ST1*^−/−^ cells incubate incubated with ATIII (two-way ANOVA with Tukey’s post hoc, **** *p* < 0.0001, n.s *p* = 0.37). Data represented as mean ± SD of 3 or 4 independent experiments (biological replicates). For images acquisition, 13 fields with 4 z-stacks were analyzed with around 2000 cells per field

Next, to understand the role of 3-*O* sulfation in the uptake of tau PFFs, the tau PFFs uptake assay was performed in absence of 3S-HS using the *HS3ST1*^−/−^ cell line, with a heparin pre-treatment condition as a positive control. Live-cell imaging showed a reduction in the number and intensity of tau-AF488 seeds puncta in *HS3ST1*^−/−^ cells (Fig. 2a). Image quantification of tau-AF488 seeds uptake (Fig. 2b) likewise revealed a significant reduction (50%) of tau uptake in the *HS3ST1*^−/−^ cell line when compared to the WT cell line, while clathrin-mediated endocytosis and macropinocytosis machineries were not halted (Fig. S2), suggesting that endocytosis is operational in the *HS3ST1*^−/−^ cells. To determine whether the remaining uptake in the *HS3ST1*^−/−^ cells is HSPGs-mediated, treatment of *HS3ST1*^−/−^ cells with heparinase was performed. A further reduction in the uptake of tau PFFs, following in a dose-response manner, was observed, with a maximum 65% inhibition at the highest heparinase concentration (0.12 U/ml). This suggest part of the uptake is facilitated by a 3-*O* sulfation-independent HSPGs-mediated uptake (Fig. S3).

To confirm that the observed phenotype is a consequence of the KO of *HS3ST1*, either a WT form of *HS3ST1* or previously reported catalytically-inactive forms of *HS3ST1* [35], were overexpressed in the *HS3ST1*^−/−^ and WT cells. The *HS3ST1* WT and mutant constructs were designed with a C-terminal mKate2 reporter, which allows for the visualization of the transfected cells. After transfection, tau-AF488 seeds were added to the cells for 24 h before live-cell imaging. Figure 2c shows representative images of *HS3ST1*^−/−^ cells transfected with either the *HS3ST1* WT protein (left panel) or reported [36] catalytically inactive E86A mutant (middle panel) or the K119A mutant (right panel). Overexpression of *HS3ST1* WT in the *HS3ST1*^−/−^ cells results in an increase of tau-AF488 puncta, when compared to tau uptake levels in *HS3ST1*^−/−^ cells overexpressing inactive forms of the protein or non-transfected *HS3ST1*^−/−^ cells. Quantification shows that *HS3ST1*^−/−^ cells overexpressing the HS3ST1 WT protein internalize tau-AF488 seeds to a level comparable to WT cells mock transfected or WT cells overexpressing with the inactive forms of the HS3ST1 protein (Fig. 2d). These results show that overexpression of HS3ST1 protein in *HS3ST1*^−/−^ cells rescues the observed decrease in tau-AF488 uptake levels, confirming the contribution of catalytic active *HS3ST1* to the internalization of aggregated tau in these cells.

In a second approach, we directly targeted HS 3-*O* sulfation. We used ATIII to bind cellular 3S-HS, hypothesizing that this would interfere with any binding of 3S-HS interactors. Here, HCT-116 WT and *HS3ST1*^−/−^ cells were pre-incubated with ATIII protein before addition of tau-AF488 seeds. Targeting cellular 3S-HS with ATIII led to a 60% reduction in the uptake of tau-AF488 PFFs. Such reduction is not observed in *HS3ST1*^−/−^ cells pre-incubated with ATIII (Fig. 2e and f). This demonstrates that when in the presence of 3-*O* sulfated HS, ATIII proteins can significantly reduce the uptake of tau PFFs.

Altogether, using both genetic and pharmacological approaches, we demonstrated that 3-*O* sulfation on HSPGs play a pivotal role in the HSPG-mediated cellular uptake of tau aggregates.

### Tau aggregates demonstrate a preferential binding to 3-*O* sulfated HS

To understand how HS 3-*O* sulfation contributes to the uptake of tau PFFs, we studied the binding of tau PFFs to HS with defined sulfation patterns.

**Fig. 3:**
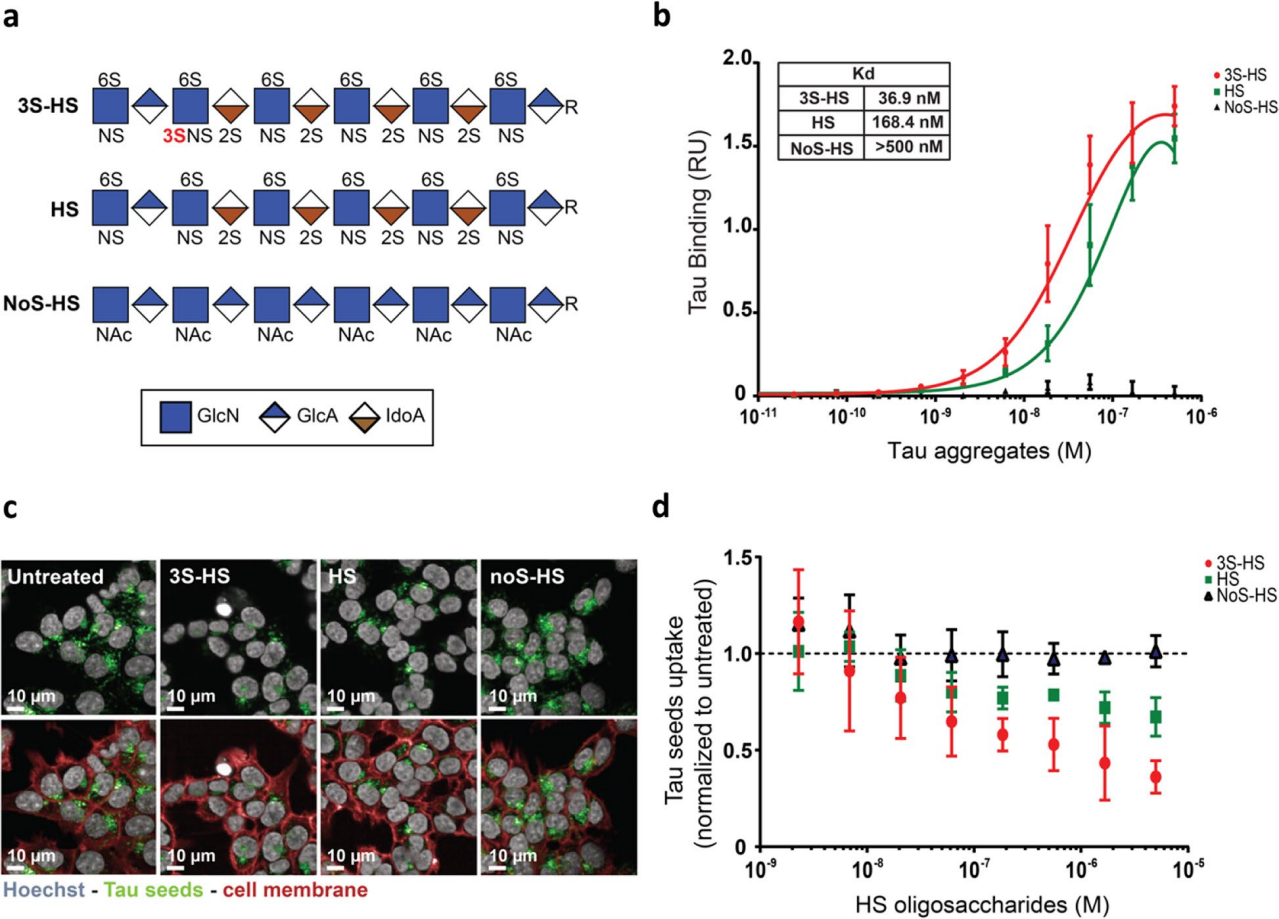
Tau PFFs bind with higher affinity to 3S-HS, resulting in a decreased uptake. **a** Structure and sulfation pattern of the synthetic heparan sulfates: *3-O* sulfated (3S-HS), non-*3-O* sulfated (HS) and non-sulfated (NoS-HS) oligosaccharides. GlcN: glucosamine, GlcA: glucuronic acid, IdoA: iduronic acid. **b** Using synthetic oligosaccharides immobilized on an ELISA plate, followed by incubation with tau PFFs in a dose-response, it is possible to observe a higher affinity of tau PFFs to the 3S-HS structure (Kd = 36.9 nM), comparing to the HS structure (Kd = 168.4 nM). As expected, tau PFFs showed no binding to the NoS-HS structure (Kd > 500 nM). Experiment was run in triplicates and data is represented as mean ± SD (**c**) Using live-cell microscopy to measure tau-AF488 uptake in HCT-116 WT cells, a reduction on the number and intensity of tau-AF488 seeds puncta can be observed after pre-treatment with either 3S-HS or HS oligosaccharides. Yet the observed decrease in tau uptake is larger after 3S-HS treatment, when compared to targeting with HS. NoS-HS treatment shows no effect on the number and intensity of tau-AF488 puncta. **d** Quantification of tau-AF488 seeds uptake by HCT-116 WT cells shows a dose-dependent increase in inhibition of tau uptake by the sulfated oligosaccharides, with 3S-HS oligosaccharide demonstrating an inhibition of 65% at the highest dose on tau seeds uptake. Data represented as mean ± SD of 3 independent experiments

Tau is a heparin binding protein and heparin can be used to target tau PFFs, thereby inhibiting their cellular uptake [15]. Here, synthetic HS oligosaccharides with (3S-HS), without 3-*O* sulfation (HS), or without any sulfation (NoS-HS) (structure depicted in Fig. 3a) were used to study the binding of tau PFFs to HS. All the structures contain 2-*O* sulfation, 6-*O* sulfation and *N*-sulfation. The presence of the 3-*O* sulfation in HS changed the affinity of tau PFFs to these structures. Specifically, the affinity of tau PFFs to HS containing one 3-*O* sulfation is 4.5-fold higher when compared to the HS oligosaccharide without (Kd = 36.9 nM and 168.4 nM respectively) (Fig. 3b). Corroborating previous literature [24, 25], tau PFFs showed no or neglectable binding (Kd > 500 nM) to the non-sulfated HS oligosaccharide. These data suggest, for the first time, that the presence of 3-*O* sulfation at the HS chain contributes the binding of tau aggregates.

Given the higher affinity of tau PFFs to synthetic 3S-HS, we studied if this difference in binding could have a functional effect on the uptake of tau PFFs. For that, we targeted tau-AF488 PFFs with the three different synthetic oligosaccharides shown in Fig. 3a prior to the uptake assay in HCT-116 WT cells. Targeting the tau PFFs with the 3S-HS oligosaccharide led to a dose-dependent inhibition of the uptake, with the highest concentration of the oligosaccharide (5 μM) leading to a 64% reduction of tau-AF488 PFFs uptake (Fig. 3c and d). High concentrations of the HS oligosaccharide also led to a reduction in uptake inhibition, yet to a lesser extent, with 5 μM of HS resulting in a 33% reduction on the uptake of tau-AF488 seeds. The NoS-HS oligosaccharide had no effect on uptake, as expected by the lack of tau PFFs binding. These results demonstrate that 3S-HS oligosaccharides are more potent at inhibiting the uptake of tau PFFs when compared to non-3-*O* sulfated HS. This indicates that the higher affinity of tau PFFs to 3S-HS also results in a higher inhibition of the tau-AF488 PFFs uptake, suggesting that the reduced uptake upon modulation of 3-*O* sulfation is a result of an altered binding of tau aggregates to HS.

## Discussion

The hypothesis of a prion-like propagation of tau pathology in the AD brain has been gaining momentum. Propagation is mediated by the release of a pathological tau seed from a donor neuron and the subsequent internalization of the seed by a receiving neuron, where it induces tau pathology by recruiting monomeric tau to an aggregated form [8, 37]. If propagation of tau pathology between neurons underlies disease progression, interruption of this process, by blocking the binding or internalization of the seeds for example, holds promise for the development of disease-modifying therapies.

HSPGs have been demonstrated to be required for the cellular uptake of tau aggregates [15]. Holmes et al. used pharmacological or genetic strategies to remove HS chains from the cell surface of cell lines, primary neurons and in vivo to show that the cellular uptake of tau PFFs is HSPG-mediated. An association between tau aggregates and HSPGs was previously shown in human AD brains, whereby immunostaining an intracellular colocalization of HSPGs with NFTs was observed [20, 38]. An outstanding question is which exact HSPGs are involved in the interaction with tau aggregates. In AD post-mortem brain tissue, agrin, glypicans and syndecans, but not perlecan, have been found in NFTs, and senile plaques [39]. Syndecans, especially the neuron predominant syndecan-3, were shown to facilitate the internalization of amyloid-β [40] and aggregated tau [41] via a clathrin-independent route in vitro.

The sulfation pattern of HS dictates the preference of a ligand to a certain HSPG. For example, ATIII [42] and herpes simplex virus 1 glycoprotein D (HSV1-gD) [43] each bind to a specific sequence of poly-sulfated residues. For tau monomers and aggregates, sulfation of HS was shown to be necessary for its interaction with HSPGs [24, 25]. The precise sulfation sequences of HS that drive the binding of tau aggregates are unknown. Elucidating those sulfation patterns that determine the interaction with aggregated tau could lead to potential intervention points to modulate HSPGs-mediated tau pathology propagation. With regard to the sulfation type involved in this interaction, 6-*O* sulfation and the *N*-sulfation of glucosamines are shown to be required for the binding, cellular uptake and seeding of aggregated tau, while the 2-*O* sulfation of IdoA is not involved [21, 23–26]. To address the contribution of 3-*O* sulfation to the cellular uptake of tau aggregates, a previous study generated KO polyclonal cell lines in HEK293T for each of the *HS3STs* isoforms and reported no impact on the uptake of tau aggregates [24]. However, the overall very limited baseline expression of *HS3STs* in HEK293 makes it not the ideal platform to interrogate the role of 3-*O* sulfation. Of note, a recent study described the contribution of 3-*O* sulfation to the binding and internalization of monomeric tau in a mouse cell line [26].

Here, we studied the role of 3-*O* sulfation of HS in the binding and cellular uptake of aggregated tau. Using synthetic HS oligosaccharides with defined sulfation patterns, we showed that tau aggregates bind to HS containing 6-*O* and *N*-sulfates and is not able to bind non-sulfated HS, in line with previous studies [23–25]. The addition of a single 3-*O* sulfation in a synthetic HS 12-mer increased the affinity of tau aggregates to this structure by 4.5-fold. The higher affinity is similar to what was observed for tau monomers [26]. The 3-*O* sulfated 12-mer also led to more binding to tau aggregates in vitro, as determined by the inhibitory effect on the internalization of tau PFFs when co-incubated in the culture medium. It is, however, still not clear whether the 3S-HS effect on tau binding is due to pure electrostatic interaction (coming for the addition of one sulfo group and, hence, one extra negative charge in the HS chain) or if it’s due to structural changes in HS.

By using a 3-*O* sulfation-deficient cell line or by neutralizing 3-*O* sulfation on the cell surface by using ATIII as a selective 3S-HS ligand, we observed a large reduction in the uptake of tau PFFs. This reduction in the 3-*O* sulfation-deficient cell line was not caused by an impairment of clathrin-mediated endocytosis or macropinocytosis and could be rescued upon overexpression of catalytically active but not inactive *HS3ST1*. Strikingly, cells overexpressing the catalytically inactive forms of HS3ST1 showed a further reduction in the uptake of tau PFFs. This could potentially be a dominant negative function of these mutations, impacting the tau PFF uptake by e.g., interfering with a 3S-HS-independent uptake component or by HS sequestration.

Treatment of the *HS3ST1*^−/−^ cells with heparinase resulted in a further reduction of the tau PFFs uptake, suggesting that at least part of the remaining uptake in these cells is HSPGs-dependent. When analysing the content of different HSs, some compounds that are products of isoforms other than HS3ST1 (compounds 12 and 13), meaning that one should not rule out other form of 3S-HS contributing to the uptake of tau. Nevertheless, because the qPCR data show no expression of other isoforms, in this study we were not able to conclude which specific isoforms are responsible for the synthesis of compounds 12 and 13. Thus, any remaining uptake can be explained by some level of 3S-HS dependent and/or independent mechanism, an incomplete digestion of HS chains by the heparinase and/or an HSPGs-independent uptake route in this cell line. Given we do not observe a plateau of the effect, using heparinase pre-treatment conditions in which a maximal, saturating HS digestion is obtained, will further reduce the uptake, suggesting that any HSPGs-independent route would be very limited to non-existing. Of note, heparin pre-treatment completely abolished the uptake of tau PFFs, suggesting that blocking the HS binding sites of tau aggregates is sufficient to inhibit their uptake.

Taken together, our study demonstrates for the first time that 3-*O* sulfated glucosamine residues in heparan sulfates take part in the interaction of HSPGs with aggregated tau and that modulation of 3-*O* sulfation alone is sufficient to significantly reduce the cellular uptake of tau PFFs.

Several studies have focused on elucidating the molecular mechanisms of cellular uptake of tau aggregates. Next to HSPGs, the low-density lipoprotein receptor-related protein 1 (LRP1) has been identified as a regulator of the uptake of aggregated tau in neurons [44]. The low-density lipoprotein receptors family is known to work in conjunction with HSPGs to mediate endocytosis [45], however the question arises as to whether LRP1 and HSPGs function mainly as co-receptors or independently to promote tau uptake. It has been previously demonstrated that amyloid-β protein is internalized in the neurons via a mechanism dependent on HSPGs and LRP1 [45]. LRP1 and HSPGs form complexes at the cell surface [46] and it was suggested that HSPGs serve as the initial binding site for the amyloid-β protein at the cell surface, where LRP1 mediates its internalization [45]. Such mechanism has not been studied for the uptake of tau protein but given the growing and convincing evidence of an HSPGs- and LRP1-dependent uptake mechanism, one can speculate that these mechanisms are not mutually exclusive and are likely to have synergistic roles in the uptake of tau aggregates. Recently the involvement of dynamin 1, actin and Rac1 proteins in the uptake of tau PFFs [47] was described. These proteins are part of core machineries involved in macropinocytosis, a pathway that has previously been proposed to mediate the uptake of tau aggregates, via HSPGs [15].

Our study provides evidence that *3-O* sulfation is at play during the internalization of aggregated tau. Interestingly, GWAS studies identified several SNPs in the *HS3ST1* [48] and *HS3ST5* [49] gene loci associated with AD. This genetic association highlights a potential role of *HS3STs* and 3-*O* sulfated heparan sulfate in AD etiology. Multiple isoforms of *HS3STs* are expressed in AD-affected brain regions [27, 29]. In the hippocampus of AD patients increased *HS3ST2* and *HS3ST4* mRNA levels have been detected and in zebrafish models *HS3ST2 was* reported to be critical for abnormal phosphorylation of tau protein [21].

According to the literature, the AD-affected regions of the brain express *HS3ST2*, *HS3ST4* and *HS3ST5* [27–29]. These regions should, thus, express so-called ATIII-type HS (a product of *HS3ST5* [50]) but also gD-type HS (a product of *HS3ST2*, *HS3ST4* and *HS3ST5* [29]). This study is based on the concept of tau as one heparin binding protein [25] and we demonstrated that loss of HS3ST1 results in a reduction of tau uptake, which seems to indicate that tau is a ligand to HS3ST1-modified HS (AT-type HS). It, however, does not demonstrate nor conclude that tau binds exclusively to this type of HS. Follow-up studies should focus on more complex platforms with different types of 3S-HS available to understand if any particular type of 3S-HS exerts a more pronounce effect in the interaction of tau or if the interaction is irrelevant of the type of 3S-HS.

The tau spreading hypothesis stipulates that a defined sequence of molecular and cellular events facilitates the spatiotemporal transsynaptic propagation. These involve, among others, the neuronal uptake, intracellular trafficking, cytosolic release, seeding of the aggregation and neuronal exit of tau aggregate species. Importantly, to understand what degree of reduction in uptake will translate to a mitigation of the propagation of the pathology, follow-up studies using neuronal in vitro as well as in vivo models of tau pathology spreading in which 3-*O* sulfation is modulated will need to be evaluated.

This work demonstrates for the first time an important contribution of 3-*O* sulfated HS to the binding of tau and subsequent HSPGs-dependent cellular internalization of aggregated tau and may promote further studies in the development of approaches targeting the HS-tau interaction to slow down the progression of tau pathology.

## Methods

### Tau fibrillation and labeling

A truncated form of the human tau protein, containing the four microtubules repeat domains (residues Q224-E372, corresponding to K18) with a P301L mutation, containing a myc tag at the C-terminus, was produced in *Escherichia coli* (Tebu-bio). To generate tau PFFs, 40 μM of K18 was mixed with 40 μM of low molecular weight (LMW) heparin (MP Biomedicals) and DTT (2 mM) in sodium acetate (100 mM) pH 7.0 buffer and incubated at 37 °C for 10 days. The mixture was centrifuged at 100,000 ´*g* at 4 °C for 1 h. The pellet was resuspended in sodium acetate (100 mM) pH 7.0 buffer and sonicated with a probe sonicator (two cycles of 10 × 1 s pulses at 20% amplitude). The characterization of fibrils generated with this protocol has been previously reported [51]. The homogeneous solution was aliquoted and stored at − 80 °C until further use. K18 fibrils (tau PFFs) were labeled with Alexa Fluor™ 488 TFP ester (Invitrogen) reactive dye according to the supplier’s instructions. To remove excess label, dialysis was performed using slide-A-Lyzer™ dialysis cassettes, with a 10 K MWCO (Thermo Fisher Scientific) in 5 l of PBS buffer at 4 °C for 48 h, with stirring at 200 RPM. Labeled tau PFFs (tau-AF488) solution was aliquoted and stored at − 80 °C until further use. Tau PFFs were freshly sonicated before use.

### Cell culture, plasmids and transfections

HCT-116 (RRID: CVCL_0291) cells in McCoy’s 5A (modified) medium with 10% FBS and 50 μg/ml of gentamicin were cultured in pre-coated poly-D-lysine 96-well black μclear plates (Fisher Scientific) (abbreviated to black 96wp). CHO-K1 (RRID:CVCL_0214) cells were grown in Ham’s F12 medium supplemented with 2 mM Glutamine and 10% FBS. The cells were maintained in a humidified atmosphere with 5% CO_2_ at 37 °C. To transfect HCT-116 cells, the sequences of wild-type *HS3ST1* and mutant *HS3ST1* (R63A, E86Q, E86A, H88A, H88F, K119A, R272A) were obtain from Moon et al. [36]. The constructs were designed to express *mKate2* at the C-terminus of the *HS3ST1* sequence. The constructs were cloned into *BamHI*/*XhoI*-digested pcDNA3.1(+) backbone by Epoch Life Science Inc. For transient overexpression of proteins, *HS3ST1*^−/−^ and WT HCT-116 cells were seeded at 12 × 10^3^ cells/well for one day. The cells were transfected using Lipofectamine 3000 reagent (Invitrogen) and the appropriate DNA construct amount (100 ng or 200 ng), following the manufacturer’s protocol, and incubated for 24 h.

### CRISPR/Cas9-mediated gene editing for the generation of *HS3ST1*^−/−^ HCT-116 clones

To generate *HS3ST1*^−/−^, two single-guide RNAs (sgRNAs) were designed using the CHOPCHOP webtool (http://chopchop.cbu.uib.no/), to target the *HS3ST1* gene (sgRNA 1: 5′-gggcgacgtgaaatacgcgg-3′; sgRNA 2: 5′-tgtgcacgtggtagaggctg-3′). The two crRNAs were produced by IDT DNA Alt-R™ S.p. Cas9 Nuclease 3NLS (I1074182) and Alt-R™ CRISPR tracrRNA (1072534) were obtained from IDT DNA. HCT-116 WT cells seeded at 16 × 10^3^ cells/well for 24 h and transfected following the manufacturer’s protocol. Briefly, crRNA (100 μM) was complexed with tracrRNA (100 μM), following assembly with recombinant Cas9 (145 nM) to generate ribonucleoproteins (RNPs). The cells were transfected with the sgRNA-tracrRNA-Cas9 RNP complex using Lipofectamine CRISPRMAX (Invitrogen). After 48 h, transfected cells were single-cell sorted into black 96wp, clones were expanded, sequenced by Sanger sequencing and selected for homozygous mutations in the *HS3ST1* gene.

### Fluorescence microscopy

HCT-116 WT cells were seeded at 12 × 10^3^ cells/well in black 96wp. To assess HSPG-dependent uptake of tau PFFs, one day after platting, cells were treated with either heparinase III (Sigma-Aldrich) at different doses (from 0.12 U/ml to 0.00094 U/ml) for 16 h or with LMW heparin (MP Biomedicals) at different doses (from 200 μg/ml to 1.6 μg/ml) for 3 h. Tau-AF488 (75 nM) was added to the culture medium and incubated for 24 h. Hoechst 33342 (200 μg/ml) and CellMask™ deep red plasma membrane stain (5 μg/ml) were added to the cells 5 min prior to live-cell imaging.

To detect 3-*O-*sulfation in HCT-116 *HS3ST1*^−/−^ and WT cells, cells were seeded at 12 × 10^3^ cells/well. The day after, cells were treated with either heparinase III (0.31 U/ml) or PBS at 37 °C for 16 h. The cells were fixed with 4% PFA in PBS for 15 min, followed by incubation with either heparin (200 μg/ml) or PBS at room temperature for 90 min with gentle shake. Afterwards, 10 μg/ml of antithrombin III (ATIII; ab62542, Abcam) was added to the cells and incubated at room temperature for 2 h, with gentle shake. Cells were washed five times with PBS followed by mouse anti-ATIII (1:1000; RRID: AB_779265) incubation at 4 °C overnight. The cells were washed three times with PBS followed by incubation with the goat anti-mouse antibody (1:10.000; RRID: AB_2536161) at room temperature for 1 h. Cells were washed three times and Hoechst 33342 (200 μg/ml) and CellMask™ deep red plasma membrane stain (5 μg/ml) were added to the cells 5 min prior to confocal fluorescence microscopy.

For tau-AF488 PFFs uptake assay in HCT-116 *HS3ST1*^−/−^ and WT, heparin (200 μg/ml) was added to the cells for 3 h. Afterwards, tau-AF488 (75 nM) was added to the culture medium and incubated for 24 h. Hoechst 3342 (200 μg/ml) and CellMask™ deep red plasma membrane stain (5 μg/ml) were added to the cells 5 min prior to live-cell imaging. For the *HS3ST1* WT and mutants’ overexpression assay, tau-AF488 (75 nM) was added to the cells 20 h after transfection and for 24 h, with the addition of Hoechst (200 μg/ml) 5 min prior to live-cell imaging. To block 3S-HS, both HCT-116 *HS3ST1*^−/−^ and WT cells were pre-treated with ATIII protein (10 μg/ml) or heparin (200 μg/ml) for 1 h30. Tau-AF488 (75 nM) was added to the culture medium and incubated for 5 h. Hoechst 3342 (200 μg/ml) and CellMask™ deep red plasma membrane stain (5 μg/ml) were added to the cells 5 min prior to live-cell imaging.

HCT-116 WT cells were seeded at 12 × 10^3^ cells/well. The day after, cells were treated with either 3-*O* sulfated HS, (3S-HS), non 3-*O* sulfated HS (HS) or non-sulfated HS (noS-HS) synthetic oligosaccharides at different doses (from 5 μM to 2.3 nM for 30 min) 2 h. Afterwards, cells were incubated with tau-AF488 seeds (75 nM) for 5 h. Hoechst 33342 (200 μg/ml) and CellMask deep red plasma membrane stain (5 μg/ml) were added to the cells 5 min prior to live-cell imaging.

For the image acquisition, Opera Phenix™ High-Content Screening System (PerkinElmer) was used. For live-cell imaging, the temperature was set to 37 °C and 5% CO_2_. A series of confocal images were acquired by imaging 13 fields per well at four planes in the z-axis at an interval of 1 μm section with a 40x water immersion objective per field. Images were analyzed using the maximum intensity projection of the image stack. Quantification of the confocal images acquired with the Opera Phenix, was conducted using the Harmony High-Content Imaging and Analysis Software v4.9 (PerkinElmer). For the quantification of tau-AF488 in transfected cell lines, regions of interest (ROIs) were defined based on the mKate2 signal, nuclei within those regions were defined as transfected cells and tau-AF488 puncta in those ROIs were defined as tau PFFs internalized by transfected cells. For quantification purposes, the number of tau-AF488 puncta inside transfected cells was normalized to the number of nuclei of transfected cells and multiplied by the mean of the tau-AF488 puncta fluorescence intensity. For the remaining uptake assays, no ROIs were defined, and uptake was quantified based on the number of tau-AF488 puncta normalized to the number of nuclei multiplied by the mean puncta intensity.

### mRNA levels quantification by qPCR

To perform the *HS3STs* expression profiles, HCT-116 cells were seeded at 12 × 10^3^ cells/well. RNA was extracted using the RNeasy Mini Kit (Qiagen) following the manufacturer’s protocol. RNA was converted into cDNA following the High-Capacity cDNA Reverse Transcription Kit (Applied Biosystems) protocol. Real-time quantitative PCR was performed using PrimeTime® Gene Expression Master Mix (IDT DNA), and analyzed by the QuantStudio™ 12 K Flex Real-Time PCR System (Applied Biosystems), using the prime-probe detection protocol. *Homo sapiens* TaqMan assays (IDT DNA) used were the following: Hs.PT.58.20018206.g (*HS3ST1*), Hs.PT.58.344017 (*HS3ST2*), Hs.PT.58.774300.g (*HS3ST3A1*), Hs.PT.58.19465218 (*HS3ST3B1*), Hs.PT.58.26274331 (*HS3ST4*), Hs.PT.58.20285766 (*HS3ST5*), Hs.PT.58.40666735 (*HS3ST6*), Hs.PT.39a.22214847 (*ACTB*), Hs.PT.58v.18759587 (*B2M*) and Hs.PT.58v.27737538 (*GUSB*). *ACTB*, *B2M* and *GUSB* are housekeeping genes and were used to normalize the data. Calibrated normalized relative quantities (CNRQ) values were calculated to the control conditions described in the results. Data is represented as mean ± SD, of a total of three independent experiments.

### Extraction of HS from the cells

HCT-116 WT, *HS3ST1*^−/−^ and CHO-K1 cells were seeded at 12 × 10^3^ cells/well. Around 4 × 10^6^ cells were collected for HS extraction. The HS extraction procedures were published previously with minor modification [52, 53]. Briefly, the cell pellet was suspended in water and proteolyzed with pronase E (10 mg:1 g (w/w), pronase E/protein) at 55 °C for 24 h to digest the protein. The digest solution was denatured at 100 °C for 10 min and centrifuged at 14000 rpm for 10 min to get the supernatant. The recovery calibrant ^13^C-labeled N-sulfo heparosan was added into the supernatant before subjecting to DEAE column purification [52]. DEAE column mobile phase A consisted of 20 mM Tris, pH 7.5 and 50 mM NaCl, and mobile phase B consisted of 20 mM Tris, pH 7.5 and 1 M NaCl. After loading the solution, the column was washed with 1.5 mL mobile phase A, followed by 1.5 mL mobile phase B to elute the HS fraction. The YM-3KDa spin column was applied to desalt the elute, and the retentate was subjected to heparin lyases digest. Before the digestion, a known amount ^13^C-labeled 3-*O*-sulfation oligosaccharide calibrants was added to the retentate [53]. The 100 μL of digestion solution containing 7.5 μL of enzymatic buffer (100 mM sodium acetate/2 mM calcium acetate buffer (pH 7.0) containing 0.1 g/L BSA), and 1.25 μL heparinase I (1.2 U/ml) and 2.5 μL heparinase II (1.2 U/m). The reaction solution was incubated at 37 °C for 5 h. Before recovering the digests from the digest solution, a known amount ^13^C-labeled non-3-*O*-sulfated disaccharide calibrants were added to the digestion solution. The HS disaccharides and tetrasaccharides were recovered by centrifugation, and the filter unit was washed twice with 200 μL of deionized water. The collected filtrates were freeze-dried before the derivatization with 2-aminoacridone (AMAC).

A total of five ^13^C-labeled 3-*O* sulfated oligosaccharide calibrants were used during the analysis of 3-*O* sulfated tetrasaccharides. These structures are oligo 1 GlcNAc-GlcA-GlcNAc6S-[^13^C]GlcA-GlcNS3S6S-[^13^C]IdoA2S-GlcNS6S-GlcA-pNP, oligo 2 GlcNAc-GlcA-GlcNS6S-[^13^C]GlcA-GlcNS3S6S-IdoA2S-GlcNS6S-GlcA-pNP, oligo 3 GlcNS6S-[^13^C]GlcA-GlcNS6S-[^13^C]IdoA2S-GlcNS3S6S-[^13^C]IdoA2S-GlcNS6S-GlcA-pNP, oligo 4 GlcNS-GlcA-GlcNS-[^13^C]IdoA2S-GlcNS3S-IdoA2S-GlcNS-GlcA-pNP and oligo 5 GlcNAc-GlcA-GlcNS-[^13^C]IdoA2S-GlcNS-IdoA2S-GlcNS3S-IdoA2S-GlcNS-GlcA-pNP. Eight ^13^C-labeled HS disaccharide calibrants were used for the analysis of non 3-*O* sulfated HS portion, and their structures were shown in a previous publication [52].

### Chemical derivatization of HS disaccharides and tetrasaccharides

The 2-Aminoacridone (AMAC) derivatization of lyophilized samples was performed by adding 10 μL of 0.1 M AMAC solution in DMSO/glacial acetic acid (17:3, v/v) and incubating at room temperature for 15 min as described previously [52]. Then 10 μL of 1 M aqueous sodium cyanoborohydride (freshly prepared) was added to this solution. The reaction mixture was incubated at 45 °C for 2 h. After incubation, the reaction solution was centrifuged to obtain the supernatant that was subjected to the LC-MS/MS analysis.

### LC-MS/MS analysis

The analysis of AMAC-labeled HS was performed on a Vanquish Flex UHPLC System (Thermo Fisher Scientific) coupled with TSQ Fortis triple-quadrupole mass spectrometry as the detector [52]. The ACQUITY Glycan BEH Amide column (1.7 μm, 2.1 × 150 mm; Waters, Ireland, UK) was used to separate di/tetra-saccharides at 60 °*C. mobile* phase A was 50 mM ammonium formate in water, pH 4.4. Mobile phase B is acetonitrile. The elution gradient as follows: 0–15 min 83–70% B, 15–30 min 70–50% B, 30–35 min 50% B, 35–45 min 83% B. The flow rate was 0.3 ml/min. On-line triple-quadrupole mass spectrometry operating in the multiple reaction monitoring (MRM) mode was used as the detector. The ESI-MS analysis was operated in the negative-ion mode using the following parameters: Neg ion spray voltage at 3.0 kV, sheath gas at 55 Arb, aux gas 25 arb, ion transfer tube temp at 250 °C and vaporizer temp at 400 °C. TraceFinder software was applied for data processing. The amount of HS was determined by comparing the peak area of native di/tetra-saccharides to each di/tetra-saccharides calibrant, and the recovery yield was calculated based on a comparison of the amount of recovery calibrant disaccharide in the samples and control, respectively. The results are represented as mean of two replicas of each cell line and the respective standard deviation (SD). The fold change between the *HS3ST1*^−/−^ and HCT-116 cells was calculated for each calibrant to easily assess the content differences.

### ELISA

To address and quantify the binding affinity of tau to different synthetic HS structures, the ELISA method was performed, following the supplier’s protocol. Briefly, biotinylated 3-*O* sulfated HS, (3S-HS), non-3-*O* sulfated HS (HS) or non-sulfated HS (NoS-HS) synthetic oligosaccharides (250 nM) were immobilized on a Pierce™ Streptavidin Coated Plates, Clear, 96-Well (Thermo Fisher Scientific) for 2 h at room temperature. The plates were blocked with 1x casein in PBS blocking buffer for 1 h at room temperature followed by incubation with sonicated tau PFFs in a dose-response (from 500 nM to 0.5 nM), for 1 h30 at room temperature. Bound tau was detected using anti-tau antibody PT76 [54] (1:2000) for 1 h30 at room temperature followed by incubation with anti-mouse HRP labeled antibody (Bio-Rad) (1:2000) for 1 h at room temperature. Signal was developed using the 1-step™ ultra TMB enzyme substrate solution (Thermo Fisher Scientific) and absorbance was measured at 450 nm using the multimode plate reader Envision (PerkinElmer).

## Supplementary Information

**Additional file 1: **Supplementary Methods. **Fig. S1.** Glycan array of antithrombin III protein showing binding to 3S-HS. **Table S1.** The results of the mass percentage (%) of each disaccharide and tetrasaccharide in the samples. **Table S2.** Recovery yield of the calibrant in the different samples. **Fig. S2.** Clathrin-mediated endocytosis and macropinocytosis in the *HS3ST1*^−/−^ cell line. **Fig. S3.** The remaining tau aggregates uptake in *HS3ST1*^−/−^ is heparinase-sensitive.

## Data Availability

The sequences of the cell lines *HS3ST1*^−/−^, designed using the CRISPR/Cas9 technology during the current study, and the corresponding paternal cell line (for comparison purposes) are available in the European Nucleotide Archive (ENA) repository with the accession number PRJEB55372 (https://www.ebi.ac.uk/ena/browser/view/PRJEB55372).
