# Supplementary material for: The 3-O sulfation of heparan sulfate proteoglycans contributes to the cellular internalization of tau aggregates
Source: BMC Mol Cell Biol. 2022 Dec 24;23:61. doi: 10.1186/s12860-022-00462-1 (PMC9789671; doi:10.1186/s12860-022-00462-1)
Supplement: Supplementary file 1 — Additional file 1: Supplementary Methods. Fig. S1. Glycan array of antithrombin III protein showing binding to 3S-HS. Table S1. The results of the mass percentage (%) of each disaccharide and tetrasaccharide in the samples. Table S2. Recovery yield of the calibrant in the different samples. Fig. S2. Clathrin-mediated endocytosis and macropinocytosis in the HS3ST1−/− cell line. Fig. S3. The remaining tau aggregates uptake in HS3ST1−/− is heparinase-sensitive. [file 12860_2022_462_MOESM1_ESM.docx]

### ADDITIONAL INFORMATION 1: SUPPLEMENTARY METHODS

**ATIII glycan array**

Printed slides were analyzed without any further modification of the surface. Alexa 488-conjugated antithrombin III (ATIII) was added at a concentration of 10 μg/mL in buffer (PBST, 20 mM Tris, 10% BSA). The sample (100 μL) were applied directly onto the surface of a single slide and covered with a microscope cover glass and then incubated in a humidified chamber for 60 min at room temperature. Slides were subsequently washed by successive rinses in (i) PBST, 20 mM Tris, 1% BSA and (ii) deionized water, dried and then immediately subjected to imaging. The images were acquired using the excitation wavelength of 488 nm on GenePix 4300 A scanner.

**Transferrin and dextran uptake assay**

Cells were seeded at 12x10^3^ cells/well in black 96wp and 24h later were incubated with Transferrin from Human Serum, Alexa Fluor™ 555 Conjugate (trf-AF555; Invitrogen) or Dextran-Rhodamine B, 10,000 MW, Neutral (dxt-Rhodamine; Invitrogen) (20 µg/ml and 400 µg/ml respectively) for 20 min at 37 °C. Hoechst 33342 (200 µg/ml) and CellMask™ deep red plasma membrane stain (5 µg/ml) were added to the cells 5 min prior to live-cell imaging, as described previously. The quantification of trf-AF555 and dxt-rhodamine uptake was based on the number of puncta normalized to the total number of nuclei multiplied by the mean puncta intensity. Data was normalized to the uptake in the WT cells condition.

**Tau-AF488 uptake assay on *HS3ST1^-/-^* cells treated with heparinase**

HCT-116 *HS3ST1^-/-^* cells were seeded at 12x10^3^ cells/well in black 96wp. One day after platting, cells were treated with heparinase III (Sigma-Aldrich) at different doses (from 0.12 U/ml to 0.00094 U/ml) for 16h. Tau-AF488 (75 nM) was added to the culture medium and incubated for 24h. Hoechst 33342 (200 µg/ml) and CellMask™ deep red plasma membrane stain (5 µg/ml) were added to the cells 5min prior to live-cell imaging. Uptake was measure using a fluorescent microscope as described before.

ADDITIONAL INFORMATION 2: SUPPLEMENTARY FIGURES
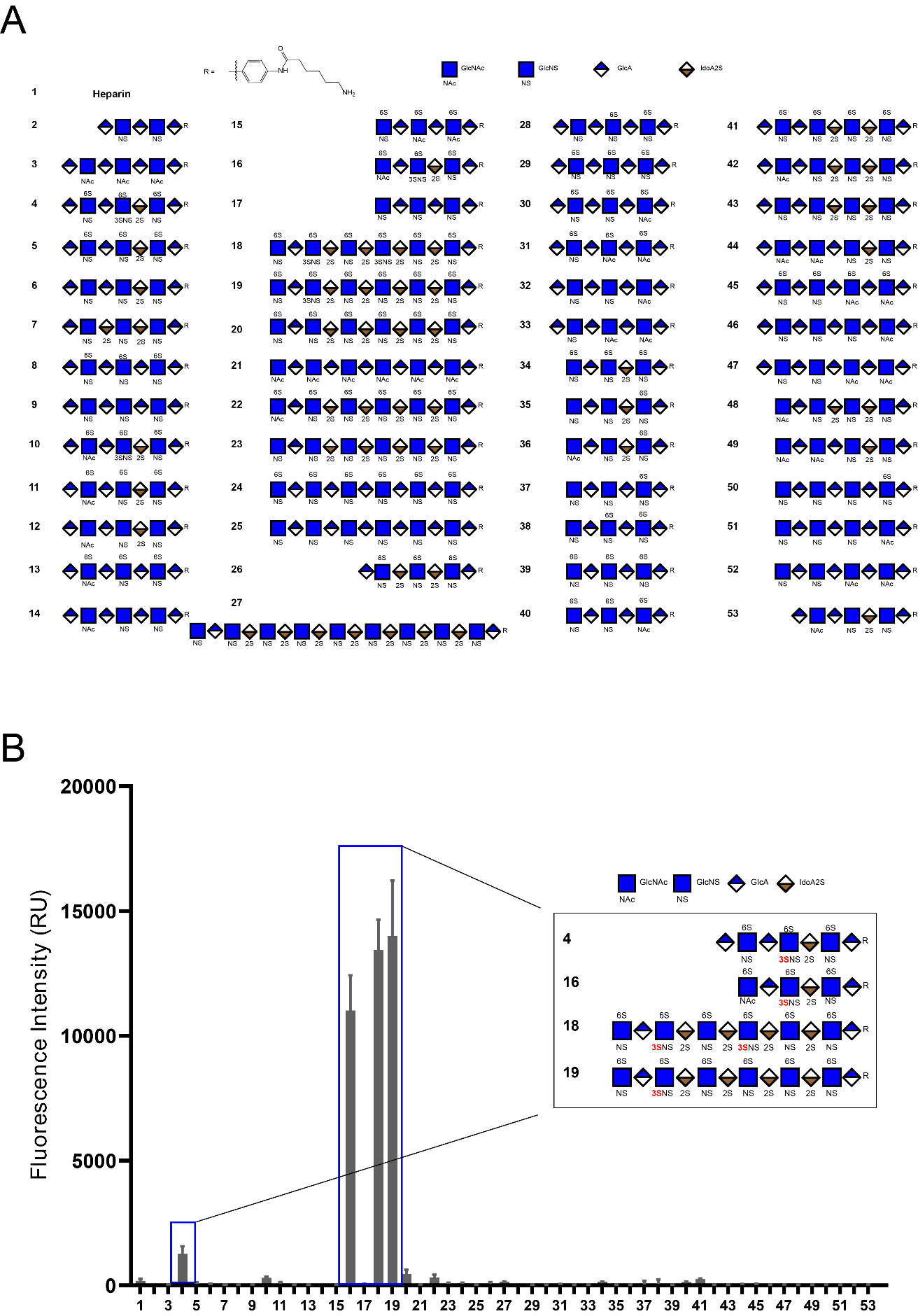


**Figure S1. Glycan array of antithrombin III protein showing binding to 3S-HS.** The glycan array method was used to assess the binding of antithrombin III (ATIII) protein to different synthetic HS structures. **(a)** The structure of the 53 oligosaccharides spotted on the glycan array. **(b)** The incubation of fluorescently labeled ATIII protein with 53 different HS synthetic structures shows higher fluorescence intensity in spots with HS containing 3-*O* sulfated HS, demonstrating the high binding selectivity of ATIII to 3-*O* sulfated HS. The fluorescence intensity data is represented as mean ± SD of 36 individual spots.

**Table S1 The results of the mass percentage (%) of each disaccharide and tetrasaccharide in the samples**

| **Di/tetra-saccharides** |  | **Amount (ng/mg)** | | | | |
| --- | --- | --- | --- | --- | --- | --- |
|  | **CHO-K1** | | ***HS3ST1*^-/-^** | | **HCT-116** | |
|  | **Mean** | **SD** | **Mean** | **SD** | **Mean** | **SD** |
| **1** △UA-GlcNAc | 185,1 | 4,5 | 65,9 | 13,6 | 46,4 | 5,4 |
| **2** △UA2S-GlcNAc | 2,7 | 0,2 | 1 | 0,4 | 0,5 | 0,0 |
| **3** △UA-GlcNAc6S | 6,2 | 0,9 | 20,8 | 8,0 | 10,3 | 1,6 |
| **4** △UA2S-GlcNAc6S | 0,1 | 0,0 | 0,2 | 0,1 | 0,1 | 0,0 |
| **5** △UA-GlcNS | 56,5 | 1,8 | 93,9 | 43,8 | 40,2 | 4,2 |
| **6** △UA2S-GlcNS | 18,1 | 0,9 | 5,7 | 2,1 | 3,4 | 0,4 |
| **7** △UA-GlcNS6S | 3,6 | 0,6 | 16,5 | 6,7 | 8,1 | 1,3 |
| **8** △UA2S-GlcNS6S | 4,9 | 0,7 | 6,2 | 1,8 | 4,4 | 1,3 |
| **9** △UA-GlcNAc6S-GlcA-GlcNS3S6S | 0 | 0,0 | 0,2 | 0,0 | 3,1 | 0,7 |
| **10** △UA-GlcNS6S-GlcA-GlcNS3S6S | 0,0 | 0,1 | 2,9 | 0,6 | 1,2 | 0,4 |
| **11** △UA-GlcNS6S-IdoA2S-GlcNS3S6S | 0 | 0,0 | 0 | 0,0 | 0 | 0,0 |
| **12** △UA-GlcNS-IdoA2S-GlcNS3S | 1,2 | 0,2 | 3,6 | 0,4 | 1,7 | 0,1 |
| **13** △UA2S-GlcNS-IdoA2S-GlcNS3S | 1,7 | 0,1 | 3,7 | 1,4 | 1,8 | 0,0 |
| **Total (ng/mg)** | 280,7 | 9,4 | 220,2 | 76,6 | 121,2 | 14,7 |

**Table S2. Recovery yield of the calibrant in the different samples**

|  |  | **Recovery yield** | | | | |
| --- | --- | --- | --- | --- | --- | --- |
|  | **CHO-K1** | | ***HS3ST1*^-/-^** | | **HCT-116** | |
|  | **Mean** | **SD** | **Mean** | **SD** | **Mean** | **SD** |
| NSK5P (ng) | 130,7 | 20,2 | 149,9 | 10,5 | 152,3 | 6,6 |
| Recovery yield (%) | 73,6 | 11,4 | 84,4 | 5,9 | 85,7 | 3,7 |


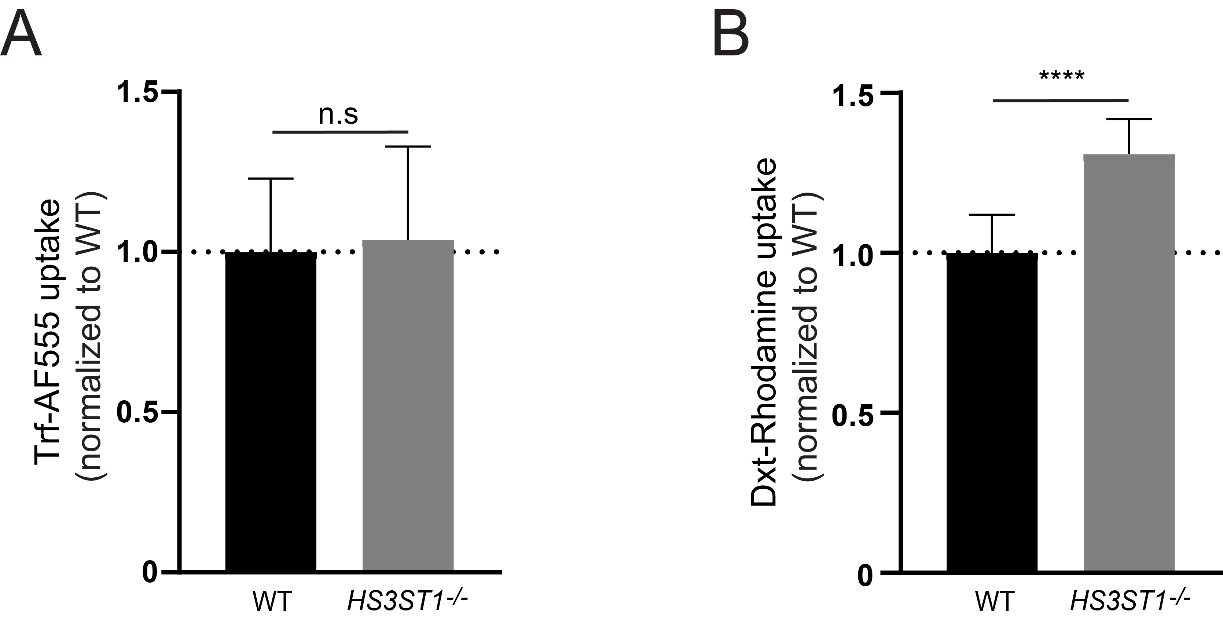


**Figure S2. Clathrin-mediated endocytosis and macropinocytosis in the *HS3ST1*^-/-^ cell line. (a)** WT and *HS3ST1*^-/-^ cells were incubated with trf-AF555 (at 20 µg/ml for 20 min), which is internalized via clathrin-mediated endocytosis. No difference in the uptake of transferrin between WT and *HS3ST1*^-/-^ cells is observed. **(b)** WT and *HS3ST1*^-/-^ cells were incubated with dxt-Rhodamine (at 400 µg/ml for 20 min), which is internalized via macropinocytosis. The uptake of dextran is 31% higher in the *HS3ST1*^-/-^ cells. (unpaired t-test was used to determine significance between WT and *HS3ST1*^-/-^ cells; n.s p=0.73, **** p<0.0001. Data represent the mean ± SD of 4 independent experiments, biological replicates).


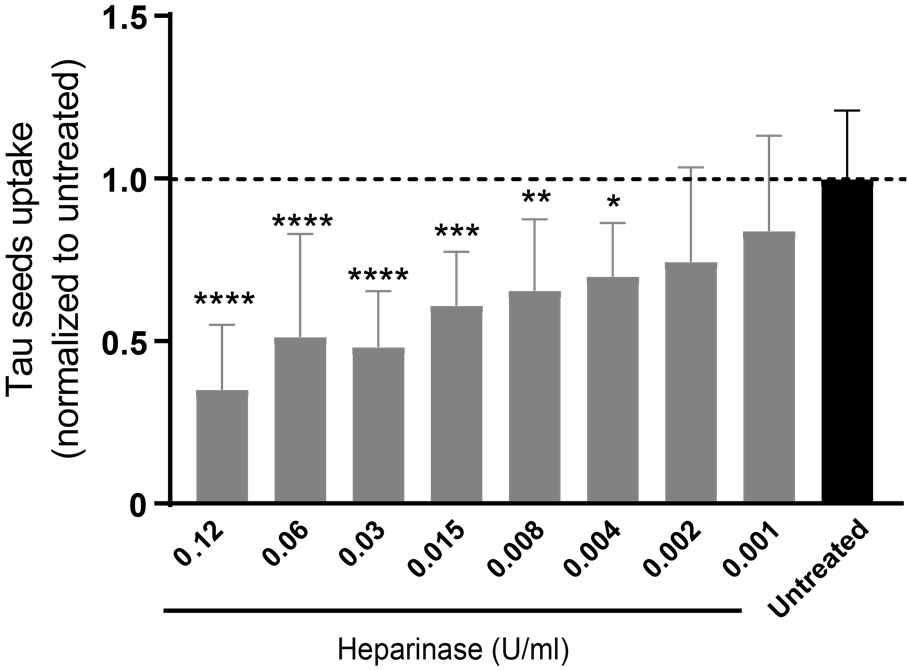


**Figure S3. The remaining tau aggregates uptake in *HS3ST1*^-/-^ is heparinase-sensitive.** *HS3ST1*^-/-^ cells were treated with heparinase in a dose-response (from 0.12 U/ml to 0.001 U/ml) to assess the uptake of tau-AF488 PFFs (24h incubation of 75 nM). Uptake quantification demonstrates a reduction of 65% in the tau PFFs uptake at the highest concentration of heparinase tested (0.12 U/ml), when compared to the untreated condition, demonstrating that a fraction of the tau PFFs uptake by *HS3ST1*^-/-^ cells is mediated by HSPGs. (Ordinary one-way ANOVA followed by Dunnet’s comparisons tests was used to determine significance between the untreated conditions and every other condition; * p<0.05, ** p<0.01, *** p<0.001, **** p<0.0001. Data is represented as the mean ± SD of 4 independent experiments, biological replicates).
